# Edge AI-Based Gait-Phase Detection for Closed-Loop Neuromodulation in SCI Mice

**DOI:** 10.3390/s26041311

**Published:** 2026-02-18

**Authors:** Ahnsei Shon, Justin T. Vernam, Xiaolong Du, Wei Wu

**Affiliations:** 1 Department of Neurological Surgery, School of Medicine, University of Louisville, Louisville, KY 40202, USA; ahnsei.shon@louisville.edu (A.S.); justin.vernam@louisville.edu (J.T.V.); 2 Kentucky Spinal Cord Injury Research Center, University of Louisville, Louisville, KY 40202, USA; dd0341@163.com; 3 Spinal Cord and Brain Injury Research Group, Stark Neurosciences Research Institute, Indiana University School of Medicine, Indianapolis, IN 46202, USA

**Keywords:** gait analysis, machine learning, vision AI, edge AI, real-time system, closed-loop neuromodulation, spinal cord injury, treadmill locomotion

## Abstract

Real-time detection of gait phase is a critical challenge for closed-loop neuromodulation systems aimed at restoring locomotion after spinal cord injury (SCI). However, many existing gait analysis approaches rely on offline processing or computationally intensive models that are unsuitable for low-latency, embedded deployment. In this study, we present a hybrid AI-based sensing architecture that enables real-time kinematic extraction and on-device gait-phase classification for closed-loop neuromodulation in SCI mice. A vision AI module performs marker-assisted, high-speed pose estimation to extract hindlimb joint angles during treadmill locomotion, while a lightweight edge AI model deployed on a microcontroller classifies gait phase and generates real-time phase-dependent stimulation triggers for closed-loop neuromodulation. The integrated system generalized to unseen SCI gait patterns without injury-specific retraining and enabled precise phase-locked biphasic stimulation in a bench-top closed-loop evaluation. This work demonstrates a low-latency, attachment-free sensing and control framework for gait-responsive neuromodulation, supporting future translation to wearable or implantable closed-loop neurorehabilitation systems.

## 1. Introduction

Gait analysis is a fundamental sensing modality in both clinical and research settings. Its applications include early detection of diseases, differentiating conditions that present with similar gait patterns, and leveraging quantitative gait metrics to anticipate future functional or clinical outcomes. Beyond diagnostic utility, gait analysis provides critical insights into the progression and severity of disorders affecting locomotion, as well as the efficacy of therapeutic interventions [1,2,3]. Neurological disorders, such as spinal cord injury (SCI), stroke, Parkinson’s disease, dementia, multiple sclerosis, amyotrophic lateral sclerosis, cerebral palsy, cerebellar ataxia, peripheral neuropathy, and muscular dystrophy, frequently result in impairments in gait and balance, leading to reduced mobility, increased fall risk, loss of functional independence, and diminished quality of life [4,5,6,7,8]. Understanding and quantifying gait abnormalities is therefore essential not only for clinical assessment and treatment planning but also for developing targeted rehabilitation strategies aimed at preserving mobility and improving overall patient outcomes.

Gait analysis can be conducted using a range of methodologies, each targeting different aspects of locomotor function. One major category uses electrical neural signals, leveraging muscular activity transmitted from the brain and spinal cord to the periphery. Invasive approaches, including nerve recordings [9,10,11] and intramuscular electromyography (iEMG) [12,13,14], provide high-fidelity information but are limited by surgical complexity and long-term stability. Non-invasive surface EMG offers a more practical alternative for routine assessment, but with reduced spatial specificity and susceptibility to motion artifacts [15,16]. A second major techniques involve wearable sensors, such as goniometers and inertial measurement units (IMUs), which directly measure joint or segmental motion [1,17,18,19,20]. However, these device-based approaches require direct attachment to the body, which can restrict natural movement, reduce user comfort, and demand a continuous power supply, limitations that make wearable sensor-based tracking particularly difficult to implement in small-animal models.

To address these limitations of body-mounted sensors, optical motion-capture systems have been widely adopted for gait analysis. Traditional marker-based systems employ reflective markers tracked by multiple cameras to reconstruct three-dimensional kinematics with high precision. Despite their effectiveness, this approach is susceptible to marker detachment, which compromises measurement accuracy, and it remains difficult to capture fine movements in anatomically small regions such as the toes or fingers during dexterous movement. These limitations have motivated the development of markerless, deep-learning-based gait analysis methods, eliminating the need for physical markers while providing robust motion estimation across a wide range of movement conditions [21,22,23].

As gait sensing technologies advance, gait analysis is being increasingly used to guide therapeutic strategies, including gait-phase-dependent neuromodulation [24,25]. Restoration of locomotor function through open-loop stimulation poses inherent limitations, as externally delivered stimulation can interfere with natural ascending and descending neural signaling [26]. In contrast, closed-loop stimulation synchronized to gait phase has gained significant attention [14,27,28]. However, the development of reliable real-time gait-phase detection algorithms traditionally requires substantial computational resources and domain expertise. Recent advances in artificial intelligence now offer new opportunities to behavior-dependent closed-loop feedback [13,29,30]. Nevertheless, for practical application, running a high-performance AI solely on a power-hungry computing system is limited, as it consumes excessive power, restricts freely moving subjects, and confines use to stationary laboratory settings. To address these limitations, we adopted a hybrid approach in which computationally intensive tasks, such as video-based pose estimation, are performed on a PC, while real-time, low-power edge AI running directly on a small-form-factor microcontroller enables low-latency gait-phase detection and significantly reduces power consumption. This architecture provides a scalable and user-friendly platform for wearable and implantable neuromodulation systems, making it suitable for practical, translational, and clinical applications.

In this study, we present a proof-of-concept AI-driven closed-loop neuromodulation system by analyzing gait patterns in sham and spinal cord-injured (SCI) mice. Using experimentally collected gait data, we constructed both a deep learning-based vision AI model with a marker-assisted approach and an on-device edge AI model. The vision AI system extracted joint angles from video data on a PC, and the edge AI system processed these outputs to classify gait phases in real time. This enabled gait-phase-dependent stimulation through the timely generation of biphasic stimulation pulses using high-resolution digital-to-analog converters (DACs) controlled by a 32-bit microcontroller, on which the edge AI is deployed, highlighting its potential for wearable or implantable applications.

The integrated system was evaluated within a bench-top environment simulating real-time conditions using recorded videos. The primary goal of this study was to validate the end-to-end system by developing and integrating its components, including vision AI for joint-angle extraction, edge AI for gait-phase detection, and gait-phase-dependent biphasic stimulation pulse generation for closed-loop neuromodulation, focusing on system-level integration rather than optimizing individual components. In the following sections, we present the gait characteristics of sham and SCI mice and describe the development and performance of the proposed vision AI and edge AI systems.

## 2. Materials and Methods

**Figure 1:**
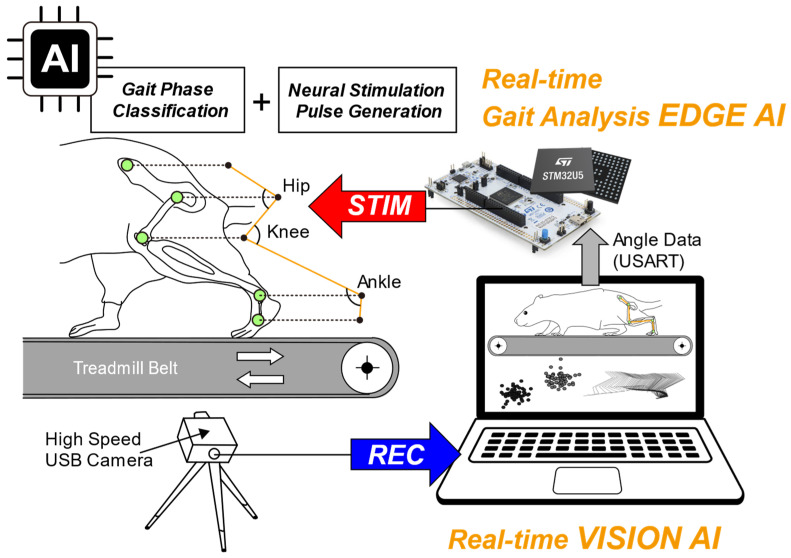
Conceptual diagram of the real-time closed-loop neuromodulation system for gait-phase-dependent neural stimulation in a SCI mouse. A high-speed universal serial bus (USB) camera records mouse locomotion on a treadmill and sends video to a laptop for vision AI-based pose estimation to extract joint coordinates (hip, knee, ankle) and calculate joint angles. The angles are transmitted via universal synchronous/asynchronous receiver/transmitter (USART) to an STM32U5-based embedded board, where the edge AI model performs real-time gait-phase classification and generates phase-specific neural stimulation patterns.

Figure 1 illustrates the conceptual framework of the proposed system for real-time, AI-based gait-phase analysis for closed-loop neuromodulation. A recorded video of a walking mouse on a motorized treadmill is processed by a hybrid AI architecture comprising vision AI and edge AI. The vision AI model running on a PC extracts predefined anatomical landmark coordinates from video data. This vision AI is integrated into custom PC software developed with the Bonsai 2.9.0 programming environment [29], which then calculates joint angles (hip, knee, and ankle) and transmits them to the edge AI model. The edge AI model deployed on a microcontroller classifies gait phases based on these joint angles into swing, stance, and abnormal phases. Firmware implemented on the microcontroller then generates precisely timed biphasic stimulation pulses to enable gait-phase-dependent neural stimulation. Detailed descriptions of the experimental setup, AI model architectures, and system implementation are provided in the following sections.

### 2.1. Treadmill Gait Analysis in Intact and SCI Mice

#### 2.1.1. Animal Preparation

To assess gait patterns, treadmill locomotion was recorded and analyzed in six mice (sham: *n* = 3; SCI: *n* = 3). Mice were housed under controlled temperature and a 12 h light/dark cycle, with ad libitum access to food and water, at the Indiana University School of Medicine animal facilities. Data were analyzed in a blinded fashion, and group identities were decoded only after analysis was completed. All experimental procedures were approved by the Institutional Animal Care and Use Committees of the Indiana University School of Medicine.

#### 2.1.2. Animal Surgery

Complete unilateral hemisection at the cervical level 4 (C4-CH), mimicking clinical Brown–Séquard syndrome, was performed using established methods with minor modifications [31]. Mice were anesthetized with a ketamine–xylazine cocktail, and a spine stabilizer was used to minimize spinal movement and ensure a left-sided lesion [32]. Following a 1 cm midline incision and ligament removal, the spinal cord at the C3–C4 vertebral level was exposed. A midline dural puncture was created using a 30 G × ½″ needle (0.3 mm × 13 mm), followed by lateral cutting with superfine iridectomy scissors. To protect the contralateral cord, a grooved 30 G × ½″ needle was inserted at the midline, and the left cord was transected along the needle using modified, blade (width: 1 mm, 10050-00, FST, Foster City, CA, USA), ensuring complete axonal transection. Contrary to the SCI group, the three mice in the sham group underwent the same surgical procedure except for the spinal cord transection.

#### 2.1.3. Video Recording of Treadmill Walking

Mice were acclimated to a motorized treadmill (LE8700RTSTH, Harvard Bioscience, New Brighton, MN, USA) for 3 days and trained to walk while the belt speed was gradually increased from 0 to 15 cm/s. After training, treadmill locomotion recordings were conducted at a constant speed of 10 cm/s, six weeks after surgery. Five anatomical landmarks (the metatarsophalangeal joint (MTP), ankle, knee, hip, and iliac crest) were selected for kinematic analysis and real-time gait-phase classification (green dots in Figure 1) based on previous locomotion research [12,33,34]. For the landmarks marking, oil-based paint (Sharpie, Atlanta, GA, USA) was applied as a marker to ensure accurate labeling for the deep-learning-based pose estimation software, DeepLabCut [21], which was used for subsequent kinematic analysis.

Anatomical landmarks were first identified by palpation and then painted directly on the skin as 2–3 mm circular dots, which improves their visibility and reduces manual labeling errors across the 200–300 frames required for DeepLabCut training, particularly for low-contrast landmarks such as the hip and iliac crest, thereby increasing overall labeling accuracy.

White paint was used for all landmarks except the MTP, which was marked in red. These contrasting colors were chosen to maximize visibility against the animal’s fur, and red was specifically used for the MTP because it contacts the treadmill belt, which is white, ensuring sufficient contrast when the foot touches the surface.

Mouse treadmill walking was recorded for 3 min per mouse using a high-speed, high-resolution color camera (Basler a2A1920-160ucPRO, Ahrensburg, Germany) positioned on the left side of the sagittal plane. Video sequences were captured at 156 frames per second (FPS) with a resolution of 1920 × 592 pixels using commercial multi-camera software (StreamPix 9, NorPix, Montreal, QC, Canada). A custom checkerboard-patterned calibration cube was used to convert pixel coordinates into millimeter-scale measurements, providing accurate spatial calibration for the subsequent kinematic analysis. For data processing, the 3 min recordings were clipped and edited in Adobe Premiere Pro 2025 (Adobe Inc., San Jose, CA, USA).

#### 2.1.4. Kinematics Analysis

A DeepLabCut network model was customized and trained to track five key anatomical landmarks of the hindlimb: the metatarsophalangeal (MTP) joint, ankle, knee, hip, and iliac crest. A total of 300 frames were labeled with the five anatomical landmarks. The network was trained using a ResNet-50 backbone architecture with a maximum of 300,000 iterations. By using the trained DeepLabCut model, the x- and y-coordinates of the anatomical landmarks were extracted from the recorded videos, along with their associated likelihood values. These coordinates were processed using custom Python scripts (Python 3.13.5, NumPy 2.2.6, SciPy 1.16.0) and MATLAB R2025 (The MathWorks Inc., Natick, MA, USA) for quantitative kinematic analysis, including spatiotemporal gait parameters. Computed parameters included joint angle range of motion (ROM), maximum and minimum joint angles, gait-cycle duration, stance and swing durations, stance duty factor, normalized joint angles, and stick diagram representations of hindlimb movements.

**Figure 3:**
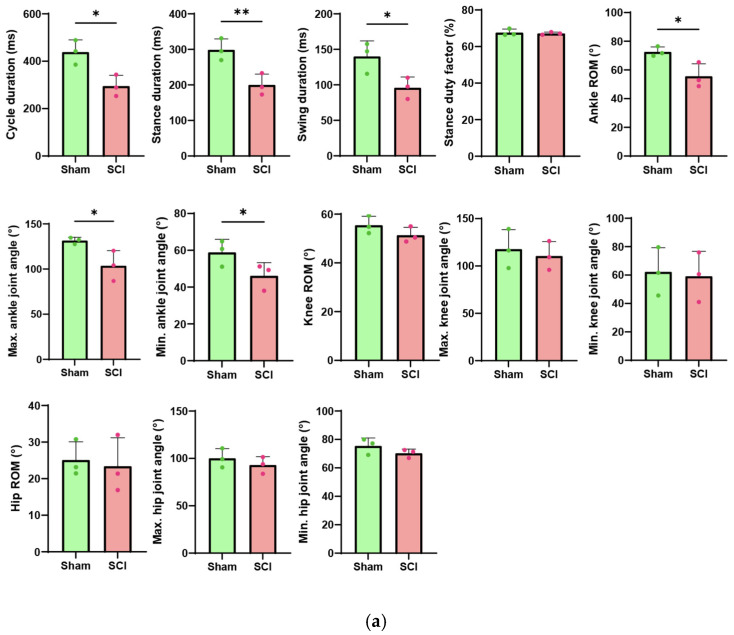

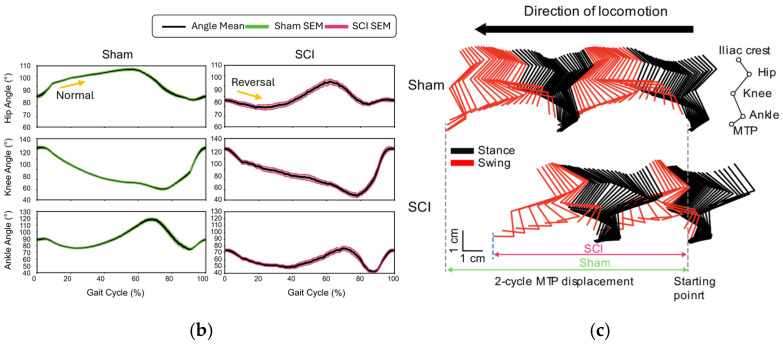
Comparison of kinematic parameters between sham and SCI groups: (**a**) Spatiotemporal gait parameters, including cycle duration, stance duration, swing duration (ms), and stance duty factor (%), and joint range of motion (ROM) for ankle, knee, and hip (degrees). Data are presented as bar graphs showing mean values with upper error bars representing +SD, with individual data points overlaid. Green circles: sham group; Magenta circles: SCI group (*n* = 3 per group), * *p* < 0.05, ** *p* < 0.01; (**b**) Representative normalized gait cycle with ankle, knee, and hip angles from one sham and one SCI mouse; (**c**) Representative stick diagrams from one sham mouse and one SCI mouse.

Statistical analysis was performed using an unpaired Welch’s *t*-test in GraphPad Prism 10.6.1, treating each animal as an independent biological replicate (*n* = 3 per group), as recommended by Lazic et al. [35]. Each data point in Figure 3a represents one animal’s mean across multiple valid gait cycles (Sham: ~156 cycles/animal; SCI: ~107 cycles/animal), preserving measurement precision while maintaining statistical independence. Any gait cycle containing frames with tracking likelihood below 0.8 was excluded to ensure data quality. This approach eliminates pseudoreplication while leveraging the central limit theorem to support approximate normality of the animal-level means. Statistical significance is indicated by asterisks in the graphs: * *p* < 0.05, ** *p* < 0.01.

### 2.2. Bench-Top Test of Vision AI and Edge AI for Closed-Loop Neuromodulation

#### 2.2.1. Vision AI-Based Extraction of Hindlimb Joint Angles

**Figure 2:**
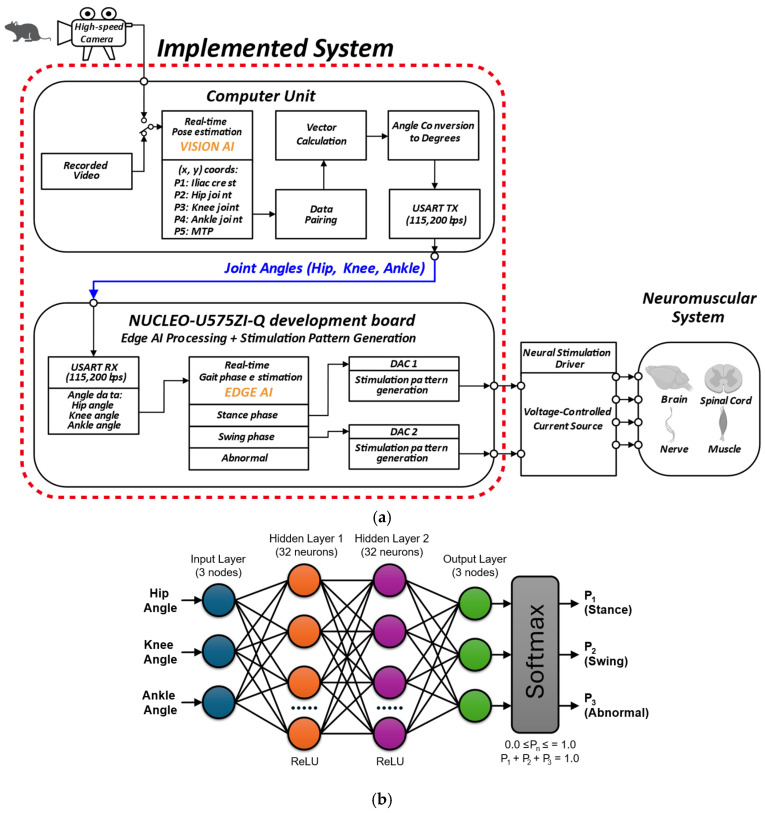
Block diagram of the integrated closed-loop neuromodulation system and edge AI model architecture: (**a**) Vision AI on the computer unit performs real-time pose estimation from recorded video. The resulting hindlimb joint angles are transmitted via USART to a microcontroller board, which performs edge AI processing and generates stimulation patterns. On the microcontroller board, the edge AI model classifies gait phases and generates stimulation trigger signals. These signals are generated through two digital-to-analog converters (DACs) to produce phase-specific neuromuscular stimulation patterns. The red dashed box indicates components implemented in this study. Images in the neuromuscular system block were created with BioRender.com and are used under a valid license; (**b**) Architecture of the lightweight edge AI neural network model, which takes three joint angles (hip, knee, ankle) as inputs and processes them through two compact hidden layers (32 ReLU neurons each). The output softmax layer produces probability estimates for stance (P_1_), swing (P_2_), and abnormal (P_3_) gait phases.

As shown in Figure 2a, gait analysis was conducted using a computer unit that processed the recorded video. A vision AI model was extracted from the trained DeepLabCut network described in Section 2.1.4. Then, the vision AI model was integrated into Bonsai 2.9.0, a visual reactive programming GUI-based tool, as described in previous research [29]. In this processing environment, the x–y coordinates of five key anatomical landmarks—the iliac crest (P1), hip joint (P2), knee joint (P3), ankle joint (P4), and metatarsophalangeal joint (MTP, P5)-were continuously extracted. These coordinates were paired to form vectors, from which joint angles were calculated using vector-based cosine methods. The resulting angles were converted to degrees, encoded in American Standard Code for Information Interchange (ASCII), and transmitted via USART at 115,200 bps through a USB-to-serial breakout board (BOB-12731, SparkFun, Niwot, CO, USA) for downstream processing.

#### 2.2.2. Edge AI Model Design and Deployment on a Microcontroller

Based on the preprocessed joint angles delivered from the PC via USART, real-time gait-phase analysis and the generation of gait-phase-dependent biphasic stimulation patterns for closed-loop neuromodulation were performed using a NUCLEO-U575ZI-Q development board, which integrates the STM32U575ZI ARM Cortex-M33 microcontroller [36]. The edge AI model developed in this study was deployed on this microcontroller.

For edge AI model development, a multilayer perceptron (MLP), a feed-forward neural network with two fully connected hidden layers (32 units each, ReLU activation) and a 3-unit softmax output layer, was implemented with 1283 trainable parameters (Figure 2b). Training input data for the model were derived from joint-angle data obtained from the coordinates described in Section 2.2.1. From six mice, data from five mice (sham, *n* = 3; SCI, *n* = 2) were used for training, while data from the one remaining SCI mouse were held out as an independent dataset for final end-to-end system evaluation.

Based on the coordinates obtained in Section 2.2.1, stance and swing phases were labeled using a previously reported pendulum-angle-based method [12,14]. Frames in which any tracked point fell below a likelihood of 0.8 due to irregular behaviors, such as twisting, moving into a corner, or running in the opposite direction, were labeled as abnormal. The training dataset was constructed by pairing joint angles extracted from DeepLabCut results with corresponding gait-phase labels (stance, swing, abnormal), totaling 140,400 samples (156 fps × 180 s × 5 mice) for edge AI model training. The training dataset intentionally retained the natural temporal distribution of gait phases, as the stance phase is inherently longer than the swing phase, thereby reflecting physiologically realistic gait patterns.

The training data were randomly shuffled and split into 80% for training and 20% for validation. The dataset consisted of time-synchronized joint-angle measurements labeled as swing, stance, or abnormal, forming a three-class classification problem. The model was trained for up to 300 epochs using the Adam optimizer (batch size = 128), with early stopping and checkpointing enabled to prevent overfitting. The trained edge AI model was evaluated and deployed on the microcontroller of the NUCLEO-U575ZI-Q development board using STM32Cube.AI 7.3.0.

#### 2.2.3. End-to-End System Latency

To investigate the end-to-end latency, defined as the time from frame acquisition to the generation of the stimulation pulse, including the entire processing pipeline on both the PC and microcontroller, two methods were employed.

On the PC side, latency was measured following a previously reported method using Bonsai software: timestamps of frame acquisition start and joint angle calculation completion were collected, and the difference between the final and initial timestamps was used to calculate PC-side latency [29].

On the NUCLEO-U575ZI-Q development board side, latency was precisely measured using a 4-channel oscilloscope (TBS2204B, Tektronix, Beaverton, OR, USA) by recording the time from the start of USART receiving, through edge AI processing, to the generation of a bipolar stimulation pulse via the DAC on the microcontroller. The total end-to-end latency was obtained by summing the PC and the NUCLEO-U575ZI-Q development board latencies.

#### 2.2.4. Gait-Phase Monitoring and Phase-Dependent Neural Stimulation

To enable real-time system-level monitoring of gait phases derived from edge AI classification outputs, two general-purpose input/output (GPIO) pins of the STM32U575ZI ARM Cortex-M33 microcontroller were configured as digital test ports. These pins were programmed to go HIGH when the corresponding phase was detected and LOW otherwise, with one pin serving as a swing phase indicator and the other as a stance phase indicator.

In addition to gait-phase monitoring, the developed system in this study is designed to generate neural stimulation patterns with precise control of stimulation parameters, including intensity, which can be adjusted across 4096 discrete levels. Two integrated 12-bit digital-to-analog converter (DAC) channels of the microcontroller were assigned for the generation of phase-specific stimulation patterns, with DAC1 outputting stimulation pulses upon detection of the stance phase and DAC2 upon detection of the swing phase. Regarding the polarity of the generated stimulation pulses, symmetrical biphasic pulses were implemented to reduce potential damage at the neural interface by limiting residual charge accumulation [37,38,39,40]. Each pulse consisted of a negative phase followed by a positive phase, each lasting 200 μs, and was generated at a frequency of 100 Hz, enabling safe and controlled neuromodulation [9,12,14]. The stimulation onset, duration, and amplitude are programmable, allowing flexible modulation of neuromuscular activation. To validate the bench-top performance of the integrated system, the two GPIO ports, representing the swing and stance phases, along with the stimulation pulses generated by DAC1 and DAC2 channels, were measured in synchronization.

## 3. Results

### 3.1. Gait Characteristics Assessed on a Treadmill in Sham and SCI Mice

Figure 3 presents spatiotemporal locomotion parameters comparing sham and SCI mice. Temporal parameters, including cycle, stance, and swing duration, are detailed in Section 3.1.1. Kinematic parameters, including range of motion (ROM) and angular excursions of the ankle, knee, and hip joints, are described in Section 3.1.2. Section 3.1.3 displays normalized gait cycles and representative stick diagrams from one sham and one SCI mouse.

#### 3.1.1. Temporal Gait Alterations in SCI Mice

As shown in the first three panels from the top left of Figure 3a, temporal parameters were significantly altered in SCI mice compared to sham controls. Notably, stance duration was markedly reduced by 33% in SCI mice, with a mean ± SD of 200.15 ± 30.50 ms compared to 298.72 ± 30.76 ms in sham mice, which was statistically significant (*p* = 0.0085). Cycle duration was similarly reduced by 33%, with SCI mice showing 295.36 ± 45.34 ms versus 438.87 ± 51.62 ms in sham mice (*p* = 0.0115), and swing duration decreased by 32%, with 95.95 ± 15.09 ms in SCI mice compared to 140.14 ± 21.83 ms in sham mice (*p* = 0.0258). Interestingly, despite these significant reductions in temporal parameters, the stance duty factor remained unchanged between groups, with 67.66 ± 1.89% for SCI mice and 67.14 ± 0.76% for sham mice (*p* = 0.3474).

#### 3.1.2. Kinematic Gait Alterations in a Representative Sham and SCI Mouse

Figure 3b shows the changes in hip, knee, and ankle joint angles over a normalized gait cycle (0–100%) for one sham mouse and one SCI mouse, illustrating gait-phase-dependent trajectories and highlighting deviations caused by spinal cord injury. Across all joints (hip, knee, and ankle), the kinematic profiles of a sham mouse closely matched previously reported gait patterns in intact rodents [41,42,43,44,45], confirming the validity of our measurement approach. The ankle joint showed characteristic patterns throughout the gait cycle, with the maximum angle at the end of stance during peak plantarflexion before toe-off, and the minimum angle at mid-swing during maximal dorsiflexion as the limb advanced forward. The hip and knee joints also displayed typical gait-phase-dependent trajectories: in sham mice, the hip angle increased during early stance before decreasing, whereas the knee angle decreased initially and then rose [41,42,43,44,45]. Building on this baseline, the representative SCI mouse exhibited clear deviations, most prominently in the ankle. The SCI mouse exhibited substantially reduced ankle angles (stance onset: 73.67°, maximum: 76.42°, minimum: 42.32°) compared with sham animals (stance onset: 88.48°, maximum: 118.30°, minimum: 74.37°), consistent with previously described patterns in injured rodent models [46]. Although ROM and peak angles of the hip and knee were largely unchanged, normalized gait-cycle trajectories revealed altered across all joints.

Notably, the SCI mouse lacked the early-stance hip elevation observed in the sham mouse; instead, the hip angle initially decreased, representing a reversal of the normal trajectory, as indicated by the yellow arrow in Figure 3b. Similar trajectory deviations occurred in the knee and ankle, indicating disrupted inter-joint coordination. These findings suggest that while some locomotor functions are preserved after unilateral cervical SCI [34], the restoration of coordinated ankle movement remains incomplete. Although temporal gait analysis revealed numerous significant differences, kinematic analysis showed minimal changes, except for the ankle angle. These observations are consistent with previous studies reporting rapid recovery of hindlimb locomotor function following cervical-level spinal cord hemisection. While forelimb function remained persistently impaired with minimal weight support, rats with C4 hemisection exhibited substantial hindlimb recovery, ultimately regaining mobility [34].

#### 3.1.3. Inter-Joint Coordination Alterations in SCI Mice

Stick diagrams were generated by connecting skeletal landmarks (iliac crest, hip, knee, ankle, and MTP) with line segments over two consecutive gait cycles, with stance phases shown in black and swing phases in red (Figure 3c). As reported in previous studies [47,48,49], the sham mouse displayed typical gait patterns with well-coordinated limb trajectories. During stance, the limb maintained an extended posture with progressive ankle plantarflexion at toe-off, while during swing, coordinated flexion occurred at all joints, achieving maximum ankle dorsiflexion at mid-swing for ground clearance. The overall trajectory envelope formed a smooth, vertically oriented pattern, reflecting efficient forward progression. These patterns were consistent with the joint angles and normalized gait-cycle peak values shown in Figure 3a,b, demonstrating reliable inter-joint coordination.

In contrast, the SCI mouse exhibited altered limb configurations throughout the gait cycle. The ankle angle was reduced during swing, particularly at swing onset, consistent with the significantly decreased maximum joint angles and swing onset peak values observed in the normalized gait cycle (Figure 3a,b). The hip was positioned more posteriorly, likely related to the reversed hip trajectory identified in Section 3.1.2. In addition, stride length was markedly shorter in the SCI mouse compared to the sham mouse. Together, these data indicate that SCI fundamentally reorganizes spatial coordination rather than merely reducing joint excursions, with stick diagrams visually corroborating the disrupted inter-joint coordination quantified in the kinematic analysis.

### 3.2. Edge AI Model Training Performance

**Figure 4:**
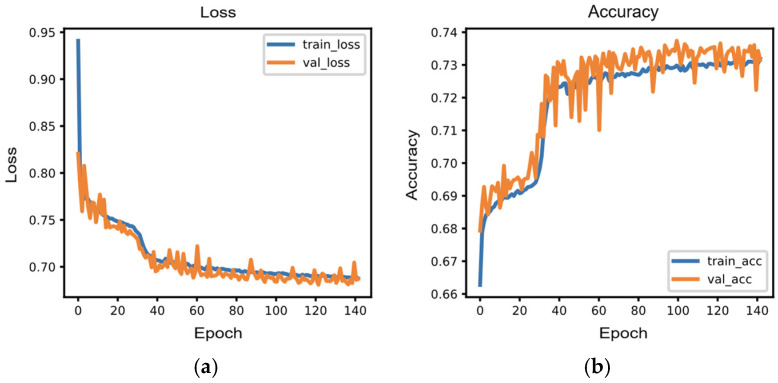
Edge AI model training performance for gait-phase classification: (**a**) Training and validation loss converged stably, enabling early stopping at epoch 141 of 300; (**b**) The model achieved 73.1% validation accuracy with minimal overfitting (73.2% training accuracy), indicating efficient and effective learning.

Training terminated at epoch 141, corresponding to 47% of the maximum 300-epoch budget, indicating efficient convergence. The model exhibited smooth learning dynamics, with training and validation losses decreasing from 0.941 and 0.820 to 0.688 and 0.687, respectively (Figure 4a). Final training and validation accuracies reached 73.2% and 73.1%, demonstrating minimal overfitting with only a 0.1 percentage point difference (Figure 4b). Performance improvements were most pronounced during the initial 30 epochs, where validation accuracy increased from 67.9% to 70.8%. Following this learning phase, the model continued gradual refinement, with validation accuracy stabilizing around 73% after epoch 80. The peak validation accuracy of 73.7% was achieved at epoch 99, after which the model maintained consistent performance until early stopping was triggered at epoch 141. This performance is comparable to previously reported gait analysis studies, which reported accuracies of 67.4% [50], 72.8% [51], and 74–80% [52]. The near-identical final loss values between training (0.688) and validation (0.687) sets, combined with the minimal accuracy gap, confirm excellent generalization capability.

The trained model was exported in HDF5 format with a file size of 47 KB (1283 trainable parameters), demonstrating compactness suitable for edge deployment. These results indicate that the lightweight MLP-based edge AI model achieved robust predictive performance for three-class gait-phase classification (swing, stance, and abnormal) while maintaining computational efficiency appropriate for real-time inference on resource-constrained platforms, potentially including microcontrollers with battery-operated implantable and wearable devices.

### 3.3. Latency from Frame Acquisition to Stimulation Pulse Generation

**Figure 5:**
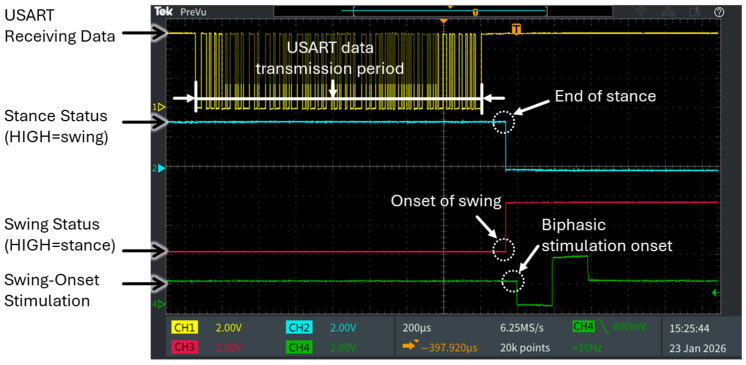
End-to-end latency on the NUCLEO-U575ZI-Q development board with the edge AI model deployed for gait-phase classification and generation of biphasic neural stimulation pulses. Latency was measured from USART data reception to edge AI-based gait-phase classification and stimulation pattern generation, totaling 1.74 ms (USART reception: 1.06 ms; gait-phase classification: 0.62 ms; biphasic stimulation generation: 0.059 ms).

The total end-to-end latency of the closed-loop system, defined as the time from video frame acquisition to the generation of a biphasic stimulation pulse, ranged from approximately 5.74 ms to 12.74 ms. The PC contribution, from frame acquisition to joint-angle calculation, ranged from 4 to 11 ms. On the microcontroller, the latency of 1.74 ms comprised three sequential stages: USART receiving (1.06 ms), gait-phase determination (0.62 ms), and DAC biphasic pulse generation (0.059 ms) (Figure 5). These results demonstrate that the microcontroller introduces only a minimal and deterministic delay, supporting precise real-time stimulation.

### 3.4. Bench-Top Evaluation of the Integrated Vision–Edge AI System

A recorded video from a single SCI mouse, which was not included in the vision AI and edge AI model training set, was used for integrated system evaluation. Hip, knee, and ankle joint angles were extracted in real-time and formatted for transmission to the edge AI model deployed on the microcontroller. The inference time of the edge AI model on the STM32U5 microcontroller was measured at 80 µs per classification, demonstrating its capability for real-time gait-phase detection with minimal latency.

**Figure 6:**
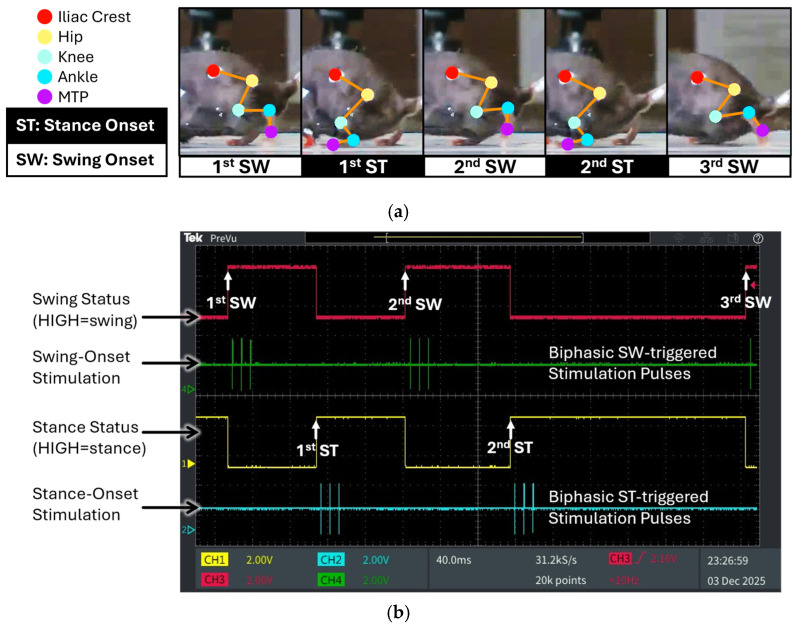
Edge AI-based gait-phase classification with monitoring signals and stimulation pulse pattern generation: (**a**) Consecutive images of a SCI mouse walking on a treadmill, showing three swing phases and two stance phases; (**b**) Oscilloscope capture showing microcontroller GPIO digital outputs for swing (magenta) and stance (yellow) detection, with DAC-generated biphasic neural stimulation pulses triggered at swing onset (green) and stance onset (cyan). Upward arrows denote temporal synchronization points corresponding to the gait-phase sequences illustrated in panel (**a**).

Figure 6 presents the output of the fully integrated vision–edge AI pipeline, illustrating the measured joint angle trajectories, corresponding gait-phase classifications, and timely generated stimulation patterns with three consecutive swing phases and two stance phases during SCI treadmill locomotion. This output demonstrates the performance of the complete system, from video input through vision AI processing to edge AI inference and microcontroller outputs. Figure 6a shows synchronized video frames with skeletal tracking overlay, illustrating the sequential gait phases (first SW, first ST, second SW, second ST, third SW) extracted by the vision AI system.

Figure 6b presents the corresponding oscilloscope traces showing real-time outputs produced by the developed edge AI model via post-processing of its softmax results. The digital waveforms generated by the microcontroller’s GPIO ports precisely align with the gait events in the video frames, as indicated by the upward arrows denoting temporal synchronization points. The system successfully classified swing and stance phases with reciprocal digital outputs: when the swing status port (magenta trace) transitioned to HIGH, the stance status port (yellow trace) remained LOW, and vice versa during stance phase detection. This reciprocal relationship confirms accurate real-time discrimination between the two primary gait phases.

In this SCI mouse, the edge AI model detected characteristic gait abnormalities. Unlike normal gait, which exhibits longer stance than swing duration, the first stance phase was shortened to approximately the same duration as the first swing phase. In contrast, the second stance phase was markedly prolonged relative to the first, resulting in an increased ankle joint angle at the onset of the third swing phase. The system accurately detected this biomechanical alteration in real-time bench-top evaluation, demonstrating sensitivity to pathological gait patterns. Additionally, the system successfully triggered phase-dependent biphasic stimulation patterns based on detected gait events. Swing-onset stimulation pulses (green trace) were generated precisely at the initiation of each swing phase, while stance-onset stimulation pulses (cyan trace) were triggered at stance transitions. Using the integrated hybrid vision–edge AI system, this study demonstrates the feasibility of edge AI-based gait-phase detection and the generation of event-driven biphasic stimulation pulses for closed-loop neuromodulation.

## 4. Discussion

This study presents a novel hybrid edge AI-based gait-phase detection system for closed-loop neuromodulation, in which vision AI extracts joint angles and edge AI classifies gait phases to generate biphasic stimulation pulses.

Locomotion recordings from both sham and SCI mice were analyzed to characterize altered gait patterns; the resulting data were used to train vision and edge AI models, which were deployed on both the PC and the small-form-factor, low-power 32-bit microprocessor of the NUCLEO-U575ZI-Q development board.

Using pre-recorded locomotion video from an SCI mouse not included in training, which simulated a real-time environment, we successfully validated the system, demonstrating the simultaneous operation of vision and edge AI for gait detection and the generation of biphasic stimulation pulses to enable closed-loop neuromodulation. The end-to-end latency, from frame acquisition to biphasic pulse generation, was measured and found to be satisfactory for real-time operation.

### 4.1. Gait Alterations in SCI Mice and Vision AI

Treadmill locomotion revealed clear differences between sham and SCI mice, with the SCI group exhibiting compensatory locomotor strategies, including altered joint angles, disrupted inter-joint coordination, and changes in spatiotemporal gait parameters. These findings are consistent with previous studies demonstrating that spinal cord injury disrupts coordinated hindlimb movements and necessitates adaptive motor strategies [53,54,55,56]. Accurate characterization of such gait deficits is essential for assessing neuromodulation efficacy and guiding rehabilitation protocol design. Traditional gait analysis methods rely on wearable devices such as EMG electrodes, IMUs, or reflective markers. Although effective, these approaches require direct skin contact and stable attachment, which can be uncomfortable in human studies and especially challenging in small-animal experiments due to reflective marker detachment or behavioral interference.

As an alternative, camera-based markerless approaches, particularly AI-driven pose estimation, enable gait capture in an unconstrained environment, improving subject comfort and reducing experimental burden. However, conventional vision-based systems generate large volumes of raw data, require extensive post-processing, and remain susceptible to occlusion and misidentification.

In the present system, the vision AI model mitigates these challenges by extracting essential anatomical landmarks and converting them into hindlimb joint angles for a real-time preprocessing step. This process can be further supported by marker-assisted labeling, in which feature points are painted with contrasting colors to improve DeepLabCut training data quality.

The painted markers provide clear visual cues and reduce labeling errors that can occur when relying solely on human visual judgment to identify kinematic key points, which are often obscured by surrounding fur or have low contrast against the background. Unlike widely used sticky reflective markers, painted markers remain securely in place throughout recording sessions, eliminating concerns about marker detachment during locomotion.

By extracting and converting raw video into compact joint angle features, the vision AI enables efficient downstream processing. Only these reduced features are transmitted to the edge AI device, minimizing bandwidth requirements and preserving data privacy through localized processing. This decentralized pipeline enhances both efficiency and translational potential.

### 4.2. On-Device Edge AI for Real-Time Closed-Loop Neuromodulation

The edge AI model performs real-time gait-phase classification, and its softmax outputs are mapped to generate precisely timed triggering signals for biphasic stimulation on a microcontroller, with processing latencies below 100 µs, enabling a robust, on-device closed-loop neuromodulation architecture. A lightweight multilayer perceptron (MLP) was selected to accommodate the computational constraints while maintaining low latency. Importantly, the integrated model—trained on combined sham and SCI datasets—generalized effectively to unseen SCI gait data, demonstrating the potential for unified models capable of handling multiple gait patterns without injury-specific training.

While the geometric method from [14] works for binary stance/swing detection, our neural network enables three-class detection (stance, swing, and abnormal), which is safer and easily expandable compared to binary detection. By detecting abnormal gait patterns due to irregular behaviors, our system prevents stimulation and can ensure clinical functional electrical stimulation (FES) safety. This is particularly important as geometric thresholds alone may struggle to detect such abnormalities. Furthermore, our framework can be easily expanded to recognize additional patterns, such as dragging, by retraining the model with more classification classes, without requiring redesign of detection logic. In contrast, geometric methods would necessitate new formulations for each pattern. Therefore, we have adopted a machine learning-based approach, which provides a practical foundation for safer FES control systems that are easily adaptable to additional detection classes. Future development should focus on adaptive, generalizable models that account for individual variability and evolving gait patterns during recovery, which will be critical for the clinical translation of closed-loop neuromodulation systems.

Notably, the novel hybrid AI pipeline was proposed, implemented, and evaluated in this study, combining vision AI for anatomical landmark detection and kinematic feature extraction with on-device edge AI for gait-phase inference to timely trigger closed-loop neural stimulation. By separating preprocessing and inference tasks across two AI modules, the system enables robust, low-latency control of gait-dependent stimulation on a low-power, on-device embedded platform. These capabilities not only demonstrate proof-of-concept for advanced neuromodulation strategies but also bridge the gap toward deployable, wearable, or implantable systems. This approach can establish a new paradigm for AI-based closed-loop neuromodulation, using two powerful AI models integrated with real-time neuromodulation.

We successfully implemented and validated the real-time edge AI model developed in this study, demonstrating its effective operation on a microcontroller. The model is capable of processing joint-angle data and generating gait-phase-triggered stimulation with low latency. On the microcontroller, total latency was 1.74 ms, of which actual computation only took 0.68 ms, while the remainder was spent transmitting the 21-byte ASCII-encoded joint-angle string at 115,200 bps. Using a higher serial rate of 921,600 bps could reduce transmission time to ~0.23 ms, lowering overall microcontroller latency to ~0.91 ms. Alternatively, encoding the joint-angle data in binary could further reduce the number of transmitted bytes, offering additional potential improvements in real-time performance.

Although the current system uses wired USART data transmission between the vision AI and edge AI modules, this link could be replaced with wireless technologies, such as medical implant communication service (MICS) [9], Bluetooth [14], Wi-Fi [57], or ultra-wideband (UWB) [58], enabling fully wireless operation for wearable and implantable closed-loop neuromodulation devices. Eliminating tethered connections would enhance patient comfort and facilitate a seamless transition toward practical wearable or implantable systems.

Furthermore, recent advances in flexible and biocompatible neural interface materials will further support the feasibility of translating closed-loop neuromodulation systems toward long-term wearable and implantable applications [59,60,61,62]. Wireless devices combined with these advanced materials can enable robust, minimally invasive, and comfortable neural interfaces for extended use in both experimental and clinical settings.

### 4.3. Limitations

Differences in gait patterns and locomotor performance between sham and SCI groups were observed, but the small sample size in each group (*n* = 3) limits statistical power and the generalizability of the findings. Larger cohorts are needed in future studies to strengthen the robustness of these conclusions. While the current experiments focus on cervical-level injuries, thoracic-level lesions should also be examined, as they typically result in more severe disruption of descending motor pathways destined for the hindlimb and may exhibit distinct compensatory gait patterns.

From a system perspective, the hybrid system combining vision AI preprocessing and edge AI inference was successfully evaluated, demonstrating that gait data can be processed and stimulation pulses generated with a minimum end-to-end latency of 5.74 ms, including 0.08 ms for edge AI processing, which is suitable for real-time closed-loop control. Also, we successfully validated the biphasic neural stimulation pulse generated by vision AI and edge AI.

The biphasic neural stimulation pulse generation method employed in this study has been validated in vivo across diverse applications, including peroneal and tibial nerve stimulation for plantarflexion and dorsiflexion control in rabbits [9], distal tibial nerve stimulation for ankle angle modulation in rats [12,13,14], and colon stimulation to assist peristaltic bowel movement in mice [63]. This demonstrated versatility across different tissues and animal models suggests that the vision–edge AI-based closed-loop framework presented here could be extended to other neuromodulation applications.

However, this evaluation relied on pre-recorded video rather than live video streams. Therefore, practical implementation will require additional experiments with freely moving subjects to confirm efficacy under actual closed-loop conditions, including real-time video acquisition, neural signal interference, and dynamic behavioral variability.

## 5. Conclusions

This study characterized locomotor differences between sham and SCI mice and demonstrated a proof-of-concept closed-loop neuromodulation system that integrates vision AI for deep-learning-based real-time pose estimation with an edge AI model deployed on a power-efficient microcontroller for gait-phase detection and triggering of precisely timed biphasic stimulation patterns at processing speeds suitable for real-time operation. The system’s low-power, locally executable AI architecture—key features of edge AI—highlights its potential for use in wearable, fully implantable, or otherwise power-limited neuromodulation devices. Furthermore, the developed system can be readily adapted for future in vivo experiments by switching from recorded video to live camera input and connecting the outputs to a voltage-controlled current source circuit and directly to the neuromuscular system. This on-device processing approach represents a step toward the development of wireless, implantable neuromodulation devices.

## Data Availability

The data presented in this study are available on request from the corresponding author.

## Abbreviations

The following abbreviations are used in this manuscript:

ASCII	American standard code for information interchange
DAC	Digital-to-analog converter
EMG	Electromyography
FES	Functional electrical stimulation
GPIO	General-purpose input/output
iEMG	Intramuscular electromyography
IMU	Inertial measurement unit
MICS	Medical implant communication service
MLP	Multilayer perceptron
ROM	Range of motion
SCI	Spinal cord injury
USB	Universal serial bus
USART	Universal synchronous/asynchronous receiver/transmitter
UWB	Ultra-wideband
